# Bacteria Induce Prolonged PMN Survival via a Phosphatidylcholine-Specific Phospholipase C- and Protein Kinase C-Dependent Mechanism

**DOI:** 10.1371/journal.pone.0087859

**Published:** 2014-01-31

**Authors:** Saskia F. Erttmann, Nelson O. Gekara, Maria Fällman

**Affiliations:** Department of Molecular Biology, Umeå Centre for Microbial Research (UCMR), Laboratory for Molecular Infection Medicine Sweden (MIMS), Umeå University, Umeå Sweden; IISER-TVM, India

## Abstract

Polymorphonuclear leukocytes (PMNs) are essential for the human innate immune defense, limiting expansion of invading microorganisms. PMN turnover is controlled by apoptosis, but the regulating signaling pathways remain elusive, largely due to inherent differences between mice and humans that undermine use of mouse models for understanding human PMN biology. Here, we aim to elucidate signal transduction mediating survival of human peripheral blood PMNs in response to bacteria, such as *Yersinia pseudotuberculosis*, an enteropathogen that causes the gastro-intestinal disease yersiniosis, as well as *Escherichia coli* and *Staphylococcus aureus*. Determinations of cell death reveal that uninfected control cells undergo apoptosis, while PMNs infected with either Gram-positive or -negative bacteria show profoundly increased survival. Infected cells exhibit decreased caspase 3 and 8 activities, increased mitochondrial integrity and are resistant to apoptosis induced by a death receptor ligand. This bacteria-induced response is accompanied by pro-inflammatory cytokine production including interleukin-8 and tumor necrosis factor-α competent to attract additional PMNs. Using agonists and pharmacological inhibitors, we show participation of Toll-like receptor 2 and 4, and interestingly, that protein kinase C (PKC) and phosphatidylcholine-specific phospholipase C (PC-PLC), but not tyrosine kinases or phosphatidylinositol*-*specific phospholipase C (PI-PLC) are key players in this dual PMN response. Our findings indicate the importance of prolonged PMN survival in response to bacteria, where general signaling pathways ensure complete exploitation of PMN anti-microbial capacity.

## Introduction

Polymorphonuclear leukocytes (PMNs) fulfill a multitude of antimicrobial functions, and interaction with bacteria and bacterial products provoke activation of a great variety of processes within these cells, including activation of chemotaxis, phagocytosis and bacterial killing. Bacteria are eliminated by PMNs, by either phagocytosis or release of toxic components [1]. Mature PMNs circulating in the blood stream have a short life span that is regulated by spontaneous apoptosis. However, decreased apoptosis is observed during inflammatory conditions, and is pivotal for the inflammatory response and resolution of infection [2]. At the site of infection, PMNs can be killed by death receptor-mediated apoptosis. PMNs express a functional FAS receptor, Fas ligand (FasL) as well as the tumor necrosis factor-α (TNFα receptors [3]. During inflammation, triggering of these receptors by TNFα or FasL expressed by infiltrating macrophages results in the activation of caspase 8 and caspase 3 [4], leading to the elimination of PMNs from the infected tissue without accumulation of necrotic cells [5].

Mature PMNs contain low numbers of mitochondria compared to other cells of the innate immunity [6]. Even so, PMN mitochondria hold a transmembrane potential and exhibit a large variety of pro-apoptotic mediators such as cytochrome c and apoptosis inducing factor [6], indicating that they play a role in regulating caspase-dependent and -independent cell death. PMNs were originally thought to have low transcriptional activity, but recent studies have shown that phagocytosis of bacteria, can lead to major changes in PMN gene expression [7]. In addition, invading bacteria may either promote or inhibit PMN apoptosis depending on the context and bacterial species [8], [9]. Although tight regulation of PMN survival is acknowledged as essential for the outcome of infection, not much is known about the signaling pathways that underlie the regulation of apoptosis in human PMNs in response to microorganisms. This is in part due to their short life span that restricts in vitro manipulations for functional analysis, e.g. knock-down via siRNA, lack of suitable cells lines and the inherent differences between mice and humans that undermine the use of mouse models for understanding human PMN biology [10]. Here we aimed to determine the impact of bacterial pathogens on the survival of human PMNs with focus on signal transduction involved in the activation of caspase 3. We identified a general mechanism for induction of PMN survival that is induced upon interaction with bacteria. The mechanism mediates a dual response including inhibition of caspase 3 activity promoting PMN survival and the production of pro-inflammatory mediators. This dual response is induced via Toll like receptor (TLR) 2 and 4 and depends on signaling via protein kinase C (PKC) and phosphatidylcholine-specific phospholipase C (PC-PLC).

## Materials and Methods

### Inhibitors, Agonists, Antibodies and Dyes

Ac-DEVD-AMC, Ac-DEVD-CHO, Ac-IETD-AMC, Ac-IETD-CHO, bisindolylmaleimide I (BIM), D609, edelfosine (*sn*-Et-18-OCH_3_), genistein, Gö 9676, rottlerin, LY294002, methyl arachidonyl fluorophosphonate (MAFP), PD98059, Ro 318220, SB202190, SP600125, staurosporine, SuperFasLigand, and U-73122 were purchased from Enzo Life Sciences (Lausen, Switzerland). Sulfasalazine was from Calbiochem (Darmstadt, Germany). MK-2206 and GDC-0068 were from ChemieTek (Indianapolis, IN; USA). CGP-53353, Gö 6983, cycloheximide and propidium iodide and human recombinant TNFα were obtained from Sigma-Aldrich (St. Louis, MO, USA). LPS-EK Ultrapure (*E. coli* K12 strain), flagellin (FLA-BS ultrapure), Pam_2_CSK_4_ and Pam_3_CSK_4_ were from InvivoGen (San Diego, CA, USA) and Kdo-lipid A (Di[3-deoxy-D-manno-octulosonyl]-lipid A) was from Avanti Polar Lipids (Delfzyl, Netherlands). RPMI and HBSS (−Ca^2+^, −Mg^2+^) were purchased from Gibco, Life Technologies (Stockholm, Sweden). Phospho-SAPK/JNK (Thr183/Tyr185), phospho-p38 MAPK (Thr180/Tyr182), phospho-p44/42 MAPK (Thr202/Tyr204), phospho-Akt (Ser473), Akt, caspase 3, PARP (46D11) antibodies, and the secondary antibodies HRP-linked anti-mouse and anti-rabbit IgG were from Cell Signaling Technology (Danvers, MA, USA), while the β-actin antibody was from Santa Cruz Biotechnology (Heidelberg, Germany). FITC (fluorescein isothiocyanate) Annexin V, and calcein-AM were purchased from BD Biosciences (San Jose, CA, USA). 1,1′,3,3,3′,3′-hexamethylindodicarbo-cyanine iodide (DiIC_1_(5)) and carbonyl cyanide 3-chlorophenylhydrazone (CCCP) were obtained from Molecular Probes, (Eugene, OR, USA).

### Bacterial Strains and Growth Conditions

*Y. pseudotuberculosis* YPIII(pIB102) strain, [11], harboring the 70 kb virulence plasmid, and the plasmid-cured strain [12] were grown over night at 26°C and 150 rpm. 1∶50 dilutions of overnight cultures with RPMI (without phenol red and HEPES) were incubated for 30 min at 26°C and 150 rpm followed by temperature shift to 37°C and 150 rpm for 1 h to induce the *Yersinia* outer protein expression. *E. coli* strain MC4100 (K12 derivate) and *S. aureus* strain Newman were grown over night at 37°C, 1∶50 diluted with RPMI (without phenol red and HEPES) and incubated for 90 min at 37°C and 150 rpm. Heat-killed *Y. pseudotuberculosis* solution was prepared by heating at 80°C for 15 min followed by 10 min at 95°C.

### Preparation and Treatment of Human PMNs

Human PMNs were isolated form whole blood of healthy donors using Polymorphprep™ (AXIS-SHIELD, Oslo, Norway) according to manufacturer’s instructions and resuspended in RPMI (without phenol red and HEPES) supplemented with 7% FCS. Concerning the experimental application 1×10^5^, 1×10^6^ or 3×10^6^ PMNs were seeded in poly-L-lysine-coated 96-well, 12-well or 6-well plates and incubated for 1 h. Thereafter PMNs were infected with bacteria at indicated multiplicities of infection (MOIs) for 30 min followed by incubation in the presence of 2 µg/ml gentamicin for 1–12 h. Exposure of PMNs to inducers of apoptosis occurred simultaneously with the addition of gentamicin to the cell culture medium. When indicated, PMNs were pretreated with appropriate inhibitors or dimethyl sulfoxide (DMSO) vehicle 1 h before infection. For each inhibitor titration of concentrations was performed to obviate cytotoxic effects on PMNs; one used concentration is indicated.

### Caspase 3 and 8 Activity Assay

At indicated time points cells were centrifuged at 250×g for 3 min followed by removal of the supernatant and destruction of cells by frost shock. Active caspase 3 in each sample was allowed to react with 16.5 µM Ac-DEVD-AMC in 1x caspase 3 assay buffer (20 mM HEPES, pH 7.4, 2 mM EDTA, 0.1% 3-[(3-cholamidopropyl) dimethylammonio]-1-propanesulfonate hydrate (CHAPS) and 5 mM dithiothreitol (DTT)). Active caspase 8 of each sample was allowed to react with 15 µM Ac-IETD-AMC in 1x caspase 8 assay buffer (20 mM HEPES, pH 7.4, 2 mM EDTA, 0.1% CHAPS, 5 mM DTT and 5% sucrose). Fluorescence was determined in a fluorescence microplate reader using excitation at 360 nm and emission detection at 460 nm for caspase 3 and excitation at 380 nm and emission detection at 460 nm for caspase 8 activity in a kinetic mode every 5 min for 1 h. Background fluorescence determined for an assay buffer control, was subtracted from each value and rate of caspase activity was calculated and indicated in rates of fluorescence units (FU). Assays were performed in triplicate.

### Annexin V Binding and PI Staining

After 12 h treatment of 1×10^6^ PMNs, cells were transferred to FACS tubes and washed with warm PBS followed by addition of 100 µl 1x annexin binding buffer (10 mM HEPES, 140 mM NaCl, and 2.5 mM CaCl_2_, pH7,4) supplemented with 5 µl FITC Annexin V and 1 µl 100 µg/ml propidium iodide and thereafter incubated at 37°C for 15 min. Subsequently, 400 µl 1x annexin binding buffer was added and samples were measured and analysed by BD™ LSR II flow cytometer and FACSDiVa software version 6.1.3, BD Biosciences, San Jose California, USA.

### Determination of Mitochondrial Potential

DiIC_1_(5) (50 nM) was added to the samples 30 min before end of 12 h incubation period. As positive control for low mitochondrial potential, cells were treated with 50 µM carbonyl cyanide 3-chlorophenylhydrazone (CCCP). Cell samples were transferred to FACS tubes and washed with warm PBS followed by addition of 500 µl 1x binding buffer. Sample fluorescence levels were measured and analysed by BD™ LSR II flow cytometer and BD FACSDiVa software version 6.1.3. For overlay histograms Flowing Software version 2.5.0 University of Turku, Finland was used.

### Interleukin (IL)-8 and TNFα Immunoassays

Concentrations of IL-8 and TNFα in cell culture supernatants were determined by the use of Quantikine® ELISA Human CXCL8/IL-8 Immunoassay and Human TNFα Immunoassay according to manufacturer’s instructions (R&D systems, Abingdon, UK).

### PMN Migration Assay

PMNs were left untreated or were treated with 10 ng/ml lipopolysaccharide (LPS) or 100 ng/ml Pam_3_CSK_4_ for 3, 6 and 12 h in RPMI supplemented with 0.5% FCS. Supernatants were collected and added to the lower well of a BD Falcon™ FluoroBlok™ 24-Multiwell Insert System (BD Biosciences). Additional PMNs were stained for 30 min with 1 µM calcein-AM in HBSS (−Ca^2+^, −Mg^2+^) supplemented with 0.5% BSA at 37°C, twice washed and added to the insert (in RPMI supplemented with 0.5% FCS). As positive control of PMN migration 10 nM N-formyl-methionyl-leucyl-phenylalanine (fMLP) was used and a 100% migration control was included. 100 ng/ml Pam_3_CSK_4_ or 10 ng/ml LPS in culture medium were used in lower well to exclude PMN migration in response to direct TLR-ligand contact. Migration was measured as fluorescence with a fluorescence microplate reader using excitation at 485 nm and emission detection at 520 nm every second minute over a two hour period at 37°C and 5% CO_2_.

### Western Blot Analysis

Protein samples were prepared by TRIzol® Reagent according to manufacturer’s instructions (Invitrogen™, life technologies). The Bradford method was used to determine the protein concentration. Equal amounts of protein were subjected to SDS-PAGE and transferred onto nitrocellulose membranes by semidry electroblotting. Membranes were blocked with 1x Roti®-Block for 1 h at room temperature followed by overnight incubation at 4°C with the primary antibody (in 20 mM Tris, 138 mM NaCl, pH 7.6, 5% (w/v) BSA, 0.1% (v/v) Tween®20). Horseradish peroxidase (HRP)-conjugated anti-mouse or anti-rabbit IgG (in 1x Roti®-Block (Roth, Karlsruhe, Germany) was used as a secondary antibody for 1 h at room temperature. Immobilon™ Western (Millipore Corporation, Billerica, USA) or Amersham™ ECL™ Western Blotting Detection Reagents (GE Healthcare, Buckinghamshire, UK) were used for detection. All experiments were performed at least three times.

### RT-qPCR

RNA was isolated via TRIzol® Reagent according to manufacturer’s instructions. For reverse transcription RevertAid H Minus First Strand cDNA Synthesis Kit from Thermo Scientific, Lithuania (EU) was used. qPCR was performed using the iQ5 real-time PCR detection system (Biorad), the PerfeCTa® SYBR Green SuperMix for iQ (Quanta Biosciences, Gaithersburg, USA) and the following primers: forward-IL-8∶5′-AGC TCT GTG TGA AGG TGC AG-3′, revers-IL-8∶5′-CTC TGC ACC CAG TTT TCC TT-3′; forward-TNFα: 5′-GCC CAG GCA GTC AGA TCA T-3′, reverse-TNFα: 5′- GCT GGT TAT CTC TCA GCT CCA-3′; forward-RPLP0∶5′-GCA ATG TTG CCA GTG TCT G-3′, reverse-RPLP0∶5′-GCC TTG ACC TTT TCA GCA A-3′. Data obtained were analysed with Bio Rad iQ5 Optical System Software version 2.0. The reference gene ribosomal protein, large, P0 (RPLP0) served for standardization of the individual PCRs. All assays were performed in duplicate.

### Statistical Analysis

Statistical analyses were performed by One-way ANOVA with Bonferroni post-test using GraphPad Prism version 5.04 for Windows, GraphPad Software, San Diego California USA.

## Results

### Avirulent and Virulent *Yersinia pseudotuberculosis* Suppress Activities of Caspase 3 and 8 in Human PMNs

**Figure 1:**
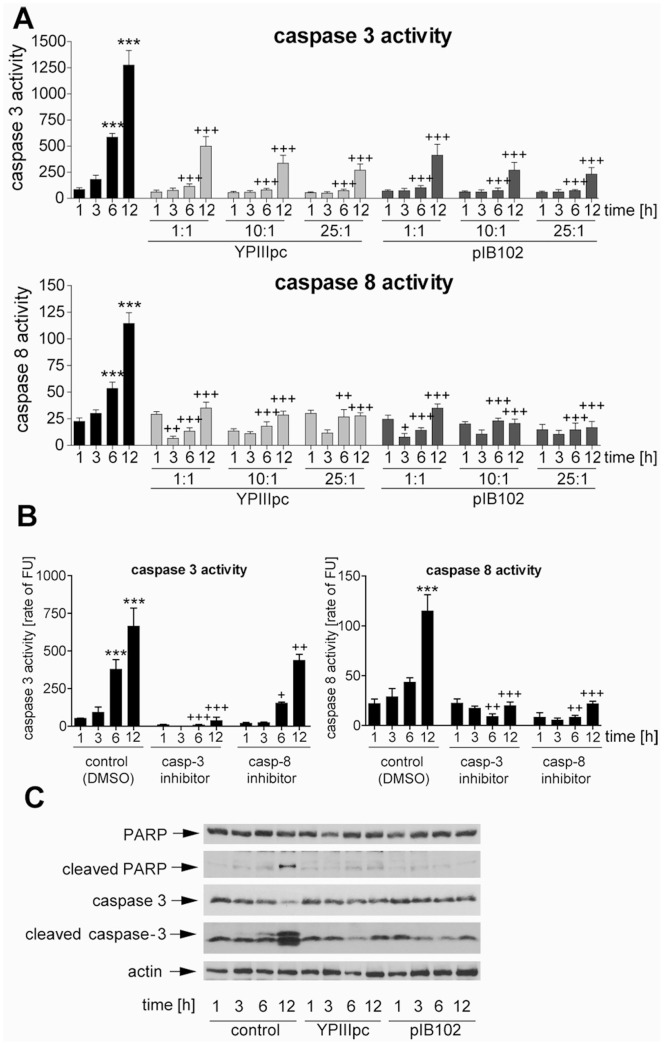
*Y. pseudotuberculosis* infection blocks caspase 3 and caspase 8 activity in human PMNs. (A) PMNs were left untreated or infected for 30 min with *Y. pseudotuberculosis* strain YPIIIpc or pIB102 at indicated MOIs followed by 1, 3, 6 and 12 h incubation in gentamicin-containing medium. Caspase 3 and 8 activities in lysates were determined using fluorometric caspase assays. The caspase activity in rate of fluorescence units (FU) is indicated. (B) PMNs were incubated with DMSO or with a specific caspase 3 or caspase 8 inhibitor for 1, 3, 6 and 12 h. Caspase 3 and 8 activities in PMN lysates were determined. The caspase activity in rate of fluorescence units (FU) is indicated. (A, B) Data are presented as mean with SEM (N = 3); ***p<0,001 compared to 1 h control; +*p*<0,05, ++*p*<0,01, +++*p*<0,001 compared to correlating time point of control panel. (C) PMNs were left untreated or infected with *Y. pseudotuberculosis* strain YPIIIpc or pIB102 at MOI 10∶1 for 30 min followed by 1, 3, 6 and 12 h incubation in gentamicin-containing medium. Cell lysates were used for Western blot analysis and probed with antibodies against caspase-3, PARP, and β-actin respectively. One experiment representative of at least three performed is shown.

To gain insight into how bacterial infections regulate apoptosis of human PMNs, we employed *Yersinia pseudotuberculosis,* a Gram-negative pathogen previously shown to modulate the cell death program in innate immune cells such as macrophages [13]. For infection we used the pathogenic strain YPIII pIB102 (pIB102) and the isogenic plasmid cured strain YPIII (YPIIIpc). The virulent pIB102 strain carries the 70 kb virulence plasmid, encoding Yersinia outer proteins (Yops) and the associated Type three secretion system that are essential for virulence including induction of apoptosis [13], [14]. Human PMNs were left untreated or infected for 30 min with different multiplicity of infection (MOI) ratios ranging from 1∶1–25∶1. Thereafter, extracellular bacteria were killed by addition of gentamicin and activities of caspase 3 and 8 in cell lysates were measured at different time points. While uninfected control cells exhibited a time-dependent increase in caspase 3 and caspase 8 activities, reflecting spontaneous apoptosis of PMNs (Fig. 1A), only low caspase 3 and 8 activities were detected in infected cells. This infection-induced inhibitory effect, obvious 3 h after infection, was independent of virulent plasmid-encoded effectors (Fig. 1A). The effect was seen at very low multiplicity of infection (MOI) (1∶1), and there were no significant differences between different infection doses with regard to the degree of blocking of the caspase activities. The specificity of the caspase 3 and 8 activity assay was confirmed by employing the caspase 3 inhibitor Ac-DEVD-CHO and the caspase 8 inhibitor Ac-IETO-CHO (Fig. 1B). Moreover, immunoblot analysis revealed pro-caspase 3 degradation and active caspase 3 in uninfected, but not in *Y. pseudotuberculosis*-infected PMNs (Fig. 1C). Cleavage of poly ADP ribose polymerase 1 (PARP1) a downstream target of caspase 3 was readily detected in uninfected PMNs after 12 h, but not in infected PMNs (Fig. 1C). In conclusion, these data show that infections with both virulent and avirulent *Y. pseudotuberculosis* mediate inhibition of caspase 3 and 8 activities resulting in increased PMN survival.

### FasL-induced Activation of Caspase 3 is Prevented in Cells Pre-infected with *Y. pseudotuberculosis*

**Figure 2:**
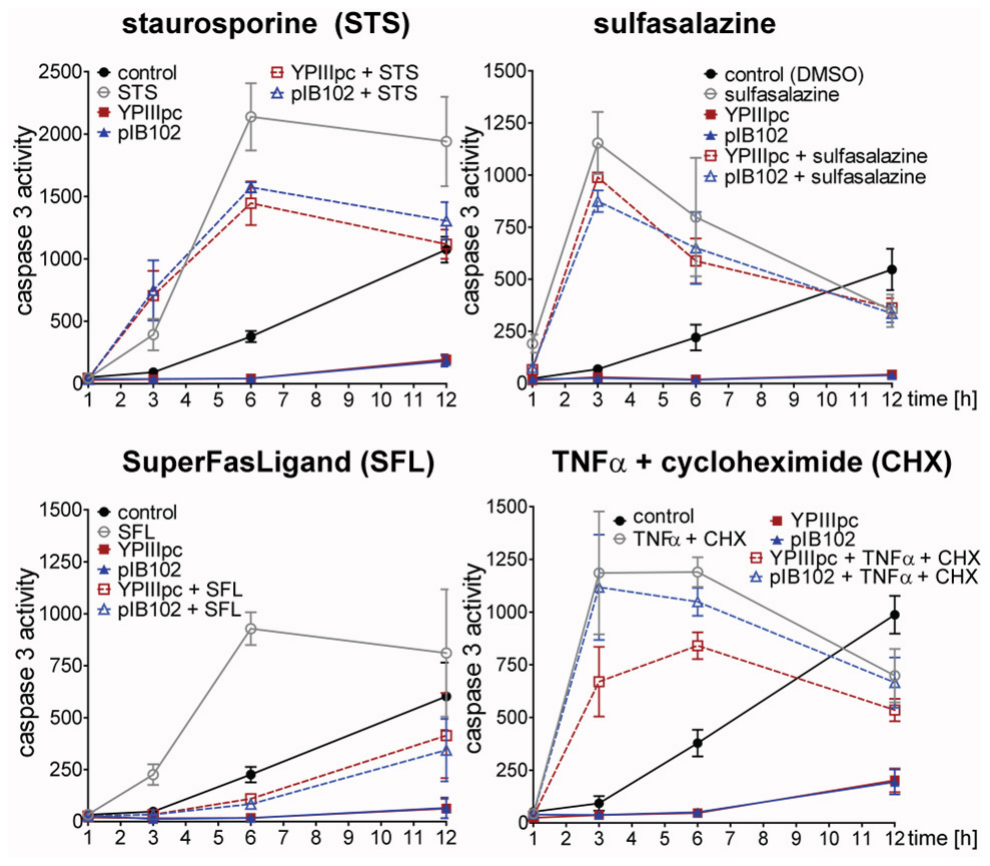
Infection with *Y. pseudotuberculosis* prevents SuperFasLigand-induced caspase 3 activation. PMNs were left untreated or infected for 30∶1 followed by addition of 2 µM STS, 1 mM sulfasalazine, 50 ng/ml SFL or 10 ng/ml TNFα and 5 µg/ml CHX and incubation for 1, 3, 6 and 12 h. Caspase 3 activity in rate of FU is indicated. Data are presented as mean with SEM (N = 4).

In view of the observed pro-survival effect by the bacteria (Fig. 1A), we next addressed whether *Y. pseudotuberculosis* infection repressed programmed cell death provoked by agents known to induce apoptosis via different mechanisms including the protein kinase inhibitor staurosporine (STS) [15], [16]; the nuclear factor κB (NFκB) inhibitor, sulfasalazine [17], [18]; the translational inhibitor, cycloheximide (CHX) [19] in combination with TNFα and SuperFasLigand (SFL). As expected, these apoptosis inducers enhanced the activity of caspase 3 in uninfected PMNs (Fig. 2). Pre-infection with *Y. pseudotuberculosis* blocked SFL-induced caspase 3 activity but not that by STS, sulfasalazine or TNFα and CHX (Fig. 2). These results were confirmed by annexin V and propidium iodide binding analyses as well as by the fluorescence dye DiIC_1_(5) for estimating mitochondrial potential (Table S1 and Fig. S1). Hence, the infection prevents FAS receptor-mediated programmed cell death whereas apoptosis induced by inhibition of different signal molecules or protein synthesis remained unaffected. Noteworthy, the latter finding indicates importance of *de-novo* protein synthesis for bacteria-induced prolonged PMN survival.

### Bacteria Induce PMN Survival and Pro-inflammatory Cytokine Responses via TLR2 or TLR4

**Figure 3:**
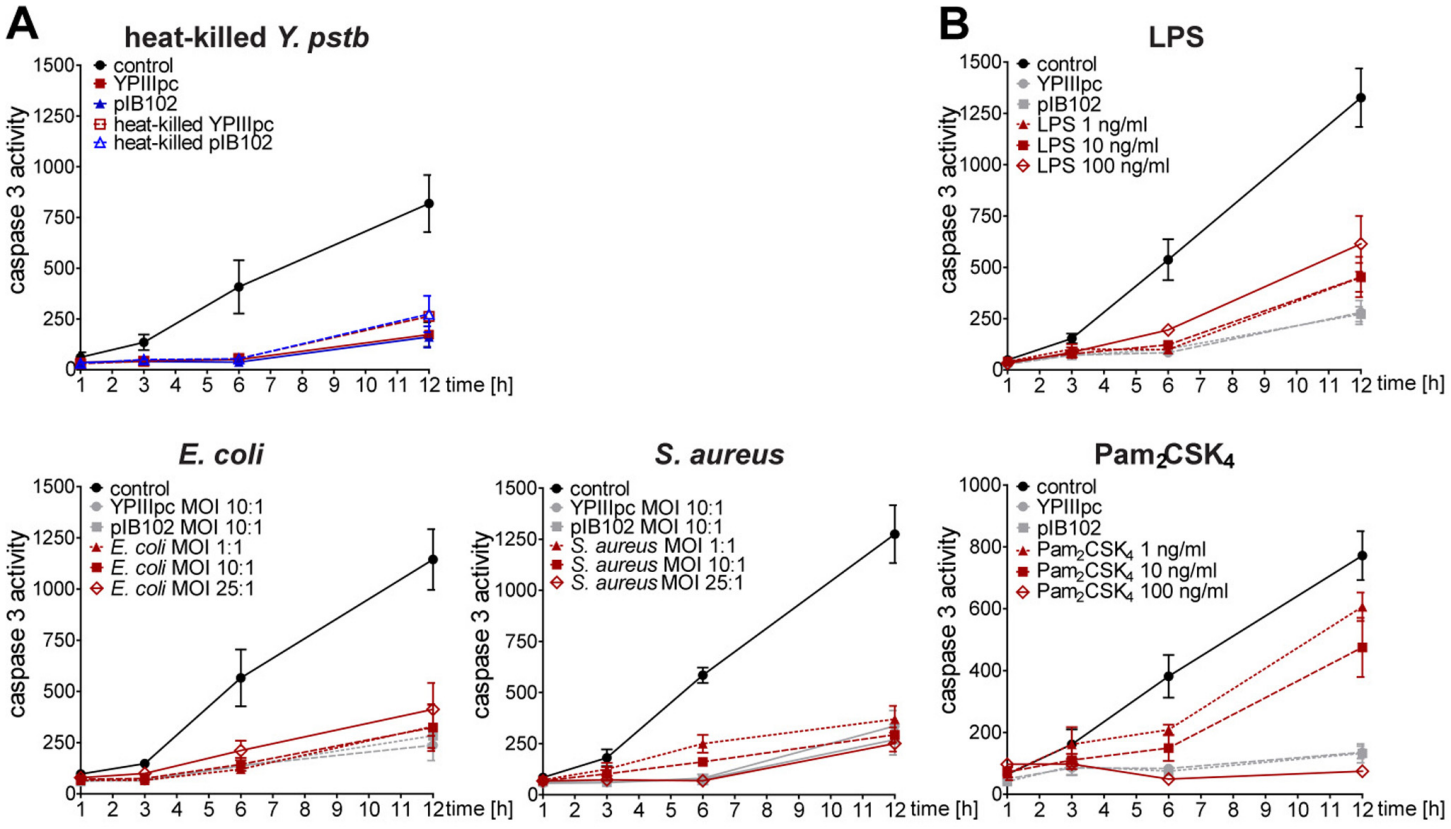
Different types of bacterial species induce PMN survival that is mediated by TLR2 and TLR4. (A) PMNs were exposed to heat-killed or live YPIIIpc or pIB102, *E. coli* MC4100 or *S. aureus* Newman at MOI 1∶1, 10∶1, 25∶1 for 30 min and further incubated for 1, 3, 6 and 12 h in gentamicin-containing medium. Untreated PMNs were used as control. Caspase 3 activity in rate of FU is indicated. Data are presented as mean with SEM (N≥3). (B) PMNs were infected with YPIIIpc or pIB102 at MOI 10∶1 or stimulated with 1, 10 and 100 ng/ml ultrapure LPS or Pam_2_CSK_4_ for 30 min followed by an incubation for 1, 3, 6 and 12 h. Caspase 3 activity in rate of FU is indicated. Data are presented as mean with SEM (N≥3).

Interaction of *Y. pseudotuberculosis* with host cells is often described as associated with secretion of Yersinia outer proteins (Yops) and their interference with host defense mechanisms [20]. However, the finding that a similar response is induced by both the YPIIIpc and pIB102 strain (Fig. 1) shows that actively secreted type three secretion factors are not responsible for the observed increase of PMN survival. Moreover, when heat-killed virulent and avirulent *Y. pseudotuberculosis* were used to stimulate PMNs, caspase 3 activity was suppressed to similar levels as that seen for live bacteria, suggesting exposure of a general bacterial structure as stimulatory agent (Fig. 3A). Subsequently we tested whether other types of bacteria exhibit an analogous property to prolong PMN survival. For this approach we used another Gram-negative species, the *E. coli* strain MC4100 as well as a Gram-positive species, the *Staphylococcus aureus* strain Newman. Measurements of caspase 3 activity demonstrated that both *E. coli* and *S. aureus* infections result in reduced apoptotic rate of PMNs in a MOI-independent manner (Fig. 3A).

Given that both Gram-positive and Gram-negative bacteria induce suppression of caspase 3 activity in PMNs, the next question arose which receptors and associated signal transduction molecules are responsible for this phenomenon. Potential candidates were the Toll-like receptors (TLRs), which are expressed by PMNs with the exception of TLR3 [21]. To investigate this possibility, PMNs were stimulated with ultrapure lipopolysaccharide (LPS) or Kdo2-lipid A as specific ligands of TLR4, with Pam_2_CSK_4_, or Pam_3_CSK_4_ as specific ligands for TLR2/TLR6 or TLR1/TLR2, and flagellin as ligand for TLR5. Both the TLR4 and TLR2 ligands induced suppression of caspase 3 activity in PMNs (Fig. 3B and Fig. S2A), while stimulation with flagellin did not suppress the time-dependent increase of caspase 3 activity (data not shown). TLR2- and TLR4-mediated inhibition of PMN apoptosis was confirmed by annexin V and propidium iodide binding assays (Table S1) as well as by mitochondrial potential analyses (Fig. S2B). Since independent activation of both receptors resulted in elevated mitochondrial potential as well as decreased caspase 3 and caspase 8 activities (Fig. 3B and Fig. S2) these data support a role of TLR2 and TLR4 in bacteria-mediated PMN survival.

**Figure 4:**
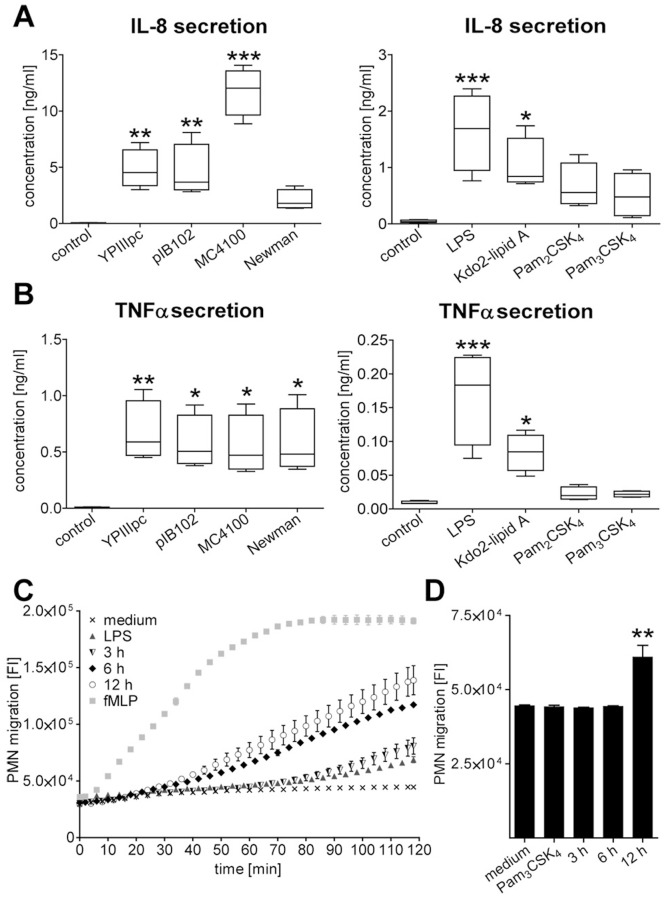
Bacterial components stimulate release of IL-8 and TNFα by PMNs triggering further recruitment of PMNs. (A, B) PMNs were infected with YPIIIpc, pIB102, *E. coli* MC4100 or *S. aureus* Newman at MOI 10∶1 for 30 min followed by incubation for additional 12 h. Concentration of IL-8 (A) and TNFα (B) in cell culture supernatants were determined by immunoassays. Data are presented as mean with SEM (N = 4); *p<0.05, **p<0.01, ***p<0.001. (C, D) In transwell plates supernatant of PMNs that were stimulated with 10 ng/ml LPS (C) or 100 ng/ml Pam_3_CSK_4_ (D) for 3, 6 or 12 h or that remained untreated, were used as attractants of calcein-AM-stained PMNs. Medium containing 10 nM fMLP was used as positive chemoattractant control while LPS- or Pam_3_CSK_4_-containing medium was used to exclude side effects. (C) Migration rate in fluorescence intensity over time (0–120 min) and (D) after 120 min is indicated. Data are presented as mean with SEM, **p<0.01. One experiment representative of three is shown.

TLR activation is associated with proinflammatory signalling and the observed prolonged life span of PMNs induced by bacteria and TLR ligands was indeed found to be accompanied by the production of pro-inflammatory mediators, measured as IL-8 and TNFα release (Fig. 4A–B). Furthermore, transwell migration assays showed migration of un-infected PMNs towards supernatants of TLR-stimulated PMNs (Fig. 4C–D). Thus, the combined TLR-mediated effects of extending the lifespan of PMNs and secretion of chemoattractants such as interleukin (IL)-8 that recruit additional PMNs to sites of infection are expected to be essential mechanisms for sustaining antibacterial assault by PMNs.

### Phosphatidylinositol 3-kinase Contributes to Survival of PMNs in Response to Bacteria

**Figure 5:**
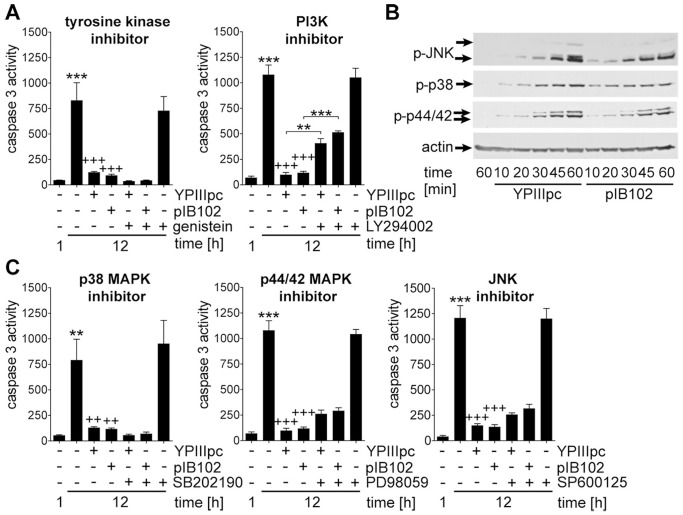
PI3K, but not tyrosine kinases contribute to bacteria-induced PMN survival. (A) PMNs were treated with 25 µM genistein or 25 µM LY294002 for 1 h before infection with YPIIIpc or pIB102 at MOI 10∶1 for 30 min followed by incubation for 1 and 12 h. Caspase 3 activity in rate of FU is indicated. Data are presented as mean with SEM (N = 3). (B) PMNs were infected with YPIIIpc or pIB102 at MOI 10∶1 for indicated periods of time. Protein extracts were subjected to Western blot analysis and probed with antibodies against phospho-JNK/SAPK, phospho-p38 MAPK, phospho-p44/42 MAPK and β-actin. One experiment representative of three performed is shown. (C) PMNs were preincubated with 5 µM SB202190, 20 µM PD98059 or 20 µM SP600125 for 1 h before infection with YPIIIpc or pIB102 at MOI 10∶1 for 30 min and further incubation for 1 and 12 h. Caspase 3 activity in rate of FU is indicated. Data are presented as mean with SEM (N = 3); (A, C) **p<0.01, ***p<0.001 compared to 1 h control unless indicated differentially, *++p*<0.01, *+++p*<0.001 compared to 12 h control.

Next we aimed to identify signaling pathways involved in bacteria-induced suppression of constitutive apoptosis in PMNs. Since genetic manipulation of this human primary cell system is not accomplishable, we employed a broad spectrum of pharmacological inhibitors. First we tested the role of tyrosine kinases and phosphoinositide 3 kinase (PI3K) using the general tyrosine kinase inhibitor genistein and the PI3K inhibitor LY294002. Genistein did not alter bacteria-induced PMN survival or caspase 3 activation (Fig. 5A) indicating that the signal transduction involved in bacteria-mediated caspase 3 inactivation does not require tyrosine kinase activity. In contrast, and in accordance with previous reports [22] granulocyte macrophage-colony stimulating factor (GM-CSF)-mediated caspase 3 suppression was reduced by genistein (Fig. S3A). Bacteria induced suppression of caspase 3 activity was abrogated by approximately 40% in the presence of the PI3K inhibitor LY294002 whereas caspase 3 activity associated with spontaneous apoptosis in uninfected PMNs remained uninfluenced by this inhibitor (Fig. 5A). In accordance with the observed dependency on PI3K, infection of untreated PMNs resulted in a time-dependent phosphorylation of the downstream PI3K target v-akt murine thymoma viral oncogene homolog (Akt) (Fig. S3B). However, the Akt-specific inhibitors MK-2206 and GDC-0068 had no effect on the reduction of caspase 3 activity triggered by bacteria (Fig. S3C).

To identify other signaling pathways downstream of TLRs involved in PMN survival, we investigated the roles of mitogen-activated protein kinase (MAPK). Both virulent and avirulent *Y. pseudotuberculosis* strains elicited a time-dependent phosphorylation of p38 MAPK, p44/42 MAPK as well as c-Jun N-terminal kinase (JNK) (Fig. 5B). However, inhibition of p38 MAPK with SB202190 did not affect bacteria-induced suppression of caspase 3 at all, while inhibition of p42/44 MAPK with PD98059 or JNK with SP600125 exerted an insignificant reduction on the bacteria-mediated effect (Fig. 5C). Since marginal elevations in caspase 3 activity in PMNs treated with these inhibitors were consistently observed in each independent experiment, participation of p44/42 MAPK and JNK in bacteria-induced caspase 3 inhibition cannot be totally excluded. Furthermore, none of the inhibitors tested influenced caspase 3 activity associated with spontaneous apoptosis in uninfected PMNs (Fig. 5C). Thus, PI3K, but not tyrosine kinases, Akt or p38 MAPK, contributes to maximal induction of PMN survival by *Y. pseudotuberculosis.*

### PKC and PC-PLC are Required for Bacteria-mediated Suppression of Caspase 3 Activity

**Figure 6:**
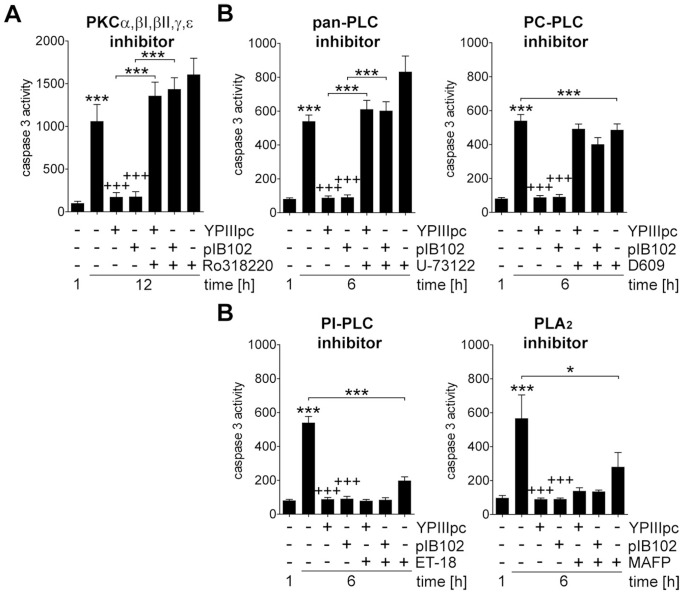
Bacteria-induced PMN survival requires PC-PLC and PKC. PMNs were treated with (A) 2 µM Ro 318220, (B) 4 µM U-73122, 10 µM Et-18-OCH_3_, 50 µM D609 or 5 µM MAFP for 1 h followed by 30 min infection with YPIIIpc or pIB102 at MOI 10∶1 and incubation for indicated time periods. Caspase 3 activity in rate of FU is indicated. Data are presented as mean with SEM (N = 4); *p<0.05, ***p<0.001 compared to 1 h control unless indicated differentially; *+++p*<0.001 compared to 12 h control.

PI3K is known to enable activation of phosphatidylinositol (3,4,5)-triphosphate (PIP_3_)-dependent kinase 1 (PDK-1) which subsequently can activate not only Akt, but also members of the protein kinase A, G, and C families. To investigate the involvement of protein kinase C (PKC), PMNs were pre-treated with different PKC inhibitors before infection with *Y. pseudotuberculosis*. Interestingly, inhibition of PKC with the general PKC inhibitor Ro 318220 [23], [24] completely abolished bacteria-induced suppression of caspase 3 activity in PMNs (Fig. 6A). To narrow down which PKC isoform influences activation of caspase 3, we tested the PKC inhibitors Gö 6976 [25], bisindolylmaleimide I (BIM) [26] and CGP 53353 [27] that block the classical isoforms α, β, γ, Gö 6983 [28] which in addition to classical PKC isoforms inhibits PKCδ and PKCζ, and the PKCδ inhibitor rottlerin [29]. However, none of these inhibitors affected bacteria-induced suppression of caspase 3 activity (Fig. S3D) thus, excluding involvement of classical PKC isoforms as well as PKCδ and ζ.

Phospholipase C (PLC) that hydrolyses phospholipids into different phosphate group derivatives and diacyl glycerol (DAG) constitutes a potential upstream mediator of PKC activation. To investigate whether PLCs participate in the signal transduction leading to prolonged PMN survival in response to bacterial infection, we employed the general PLC inhibitor U-73122 [30]. Inhibition of PLC with this inhibitor completely abrogated the suppressive effect by *Y. pseudotuberculosis* (Fig. 6B). The level of caspase 3 activity in PLC-inhibited infected PMNs was similar to that in uninfected PMNs indicating a role for PLC in bacteria-induced suppression of caspase 3 activity. For further specification, which PLC isoform is involved in the reduction of caspase 3 activity by bacteria, we used the phosphatidylinositol (PI)-specific PLC inhibitor edelfosine (Et-18-OCH_3_) [31] and the phosphatidylcholine (PC)-specific PLC inhibitor D609 [32]. Interestingly, inhibition of PC-PLC abrogated the *Y. pseudotuberculosis*-induced repression of caspase 3 activity (Fig. 6B). In contrast, inhibition of PI-PLC did not affect the bacteria-mediated suppressive effect (Fig. 6B). In addition, we also tested methyl arachidonyl fluorophosphonate (MAFP) that selectively inhibits cytosolic and Ca^2+^-independent phospholipase A_2_ (PLA_2_) that catalyse the release of arachidonic acid, a precursor of active compounds such as prostaglandins and leukotrienes [33]. However, there was no effect on bacteria-induced inhibition of caspase 3 activity, neither by blocking Ca^2+^-independent PLA_2_ nor cytosolic PLA_2_ (Fig 6B). It is noteworthy, though, that both the PI-PLC and the PLA_2_ inhibitor reduced the level of spontaneous apoptosis in uninfected PMNs. Taken together, these data provide evidence for a dominant role of PC-PLC and PKC in the regulation of suppression of PMN’s spontaneous apoptosis by bacteria.

### PC-PLC and PKC Mediate a General, Dual Response of Cytokine Release and Prolonged PMN Survival

**Figure 7:**
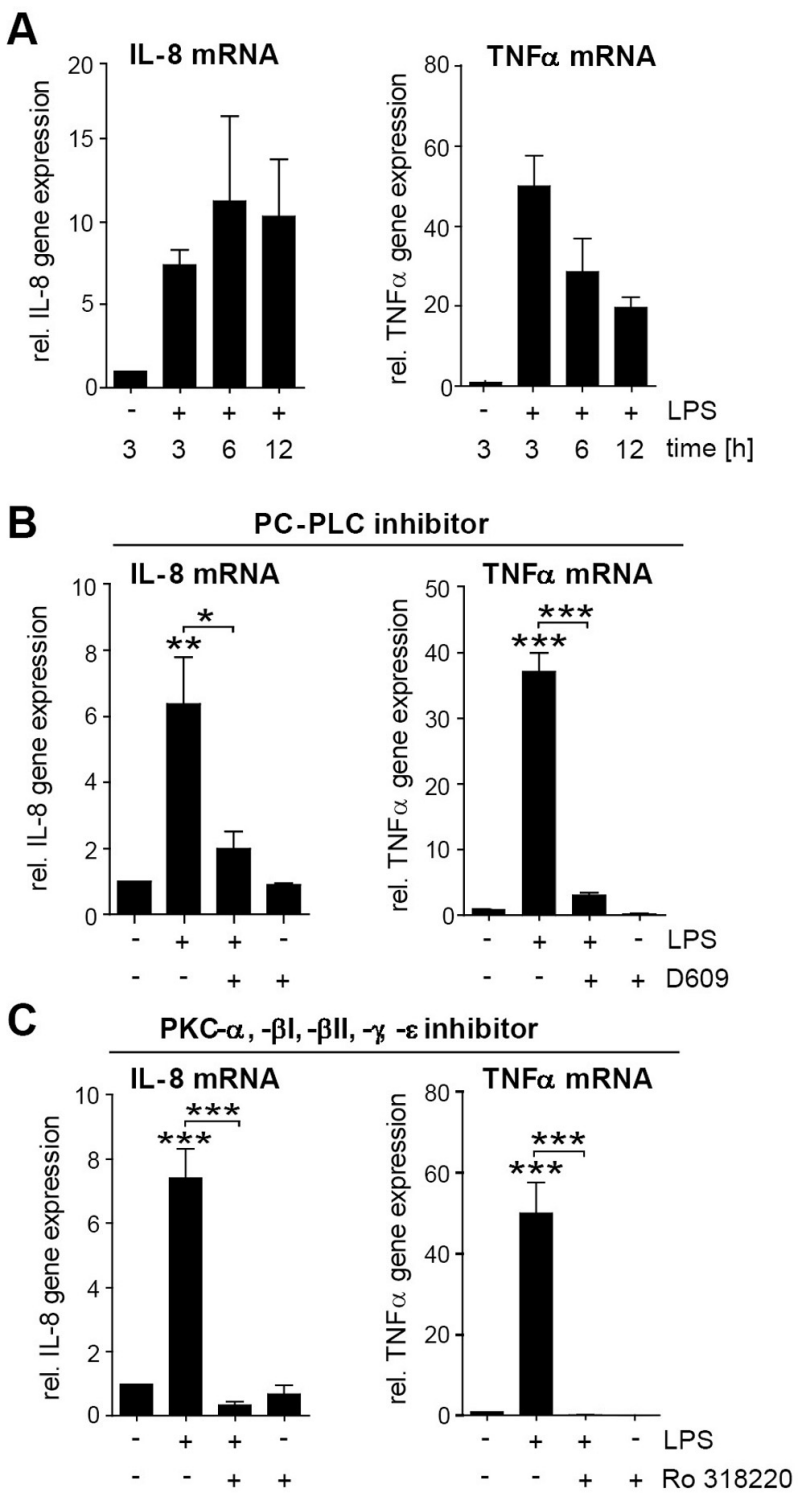
TLR-mediated induction of IL-8 and TNFα gene expression requires PC-PLC and PKC. (A) PMNs were treated with 10 ng/ml LPS for 3–12 h or with (B) 2 µM Ro 318220 or (C) 50 µM D609 for 1 h followed by LPS stimulation for 3 h. IL-8 and TNFα gene expression normalized to RPLP0 and relative to untreated/vehicle-treated PMNs is indicated (N = 3); ***p<0.001, **p<0.01, *p<0.05.

**Figure 8:**
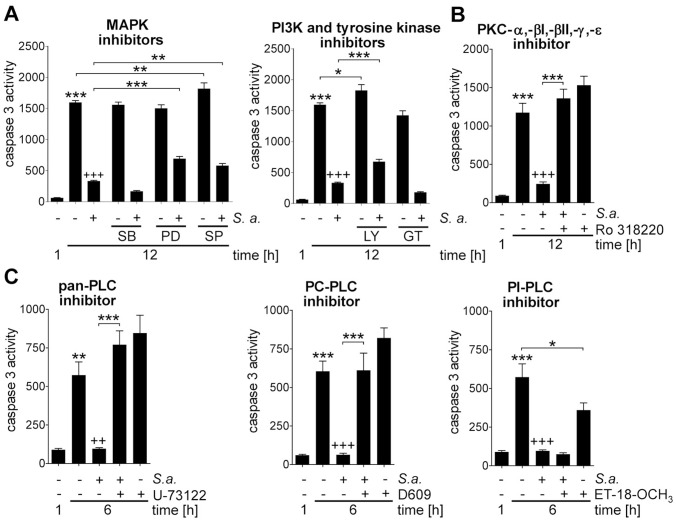
*S. aureus* induces prolonged PMN survival via PC-PLC, PI3K and PKC. PMNs were pretreated with (A) 5 µM SB202190 (SB), 20 µM PD98059 (PD), 20 µM SP600125 (SP), 25 µM genistein (GT), 25 µM LY294002 (LY), (B) 2 µM Ro 318220, (C) 4 µM U-73122, 10 µM Et-18-OCH_3_ or 50 µM D609 for 1 h followed by 30 min infection with *S. aureus* strain Newman at MOI 10∶1 and an additional incubation for indicated time points in gentamicin-containing medium. Caspase 3 activity of cell lysates in rate of FU was determined. Data are presented as mean with SEM (N = 4); *p<0.05, **p<0.01, ***p<0.001 compared to 1 h control unless indicated differentially; *++p*<0.01, *+++p*<0.001 compared to 12 h and 6 h control respectively.

Given that there is a dual response to bacteria distinguished by PMN survival and release of proinflammatory mediators, we next investigated whether PC-PLC and PKC also play a role in bacteria-induced induction of IL-8 and TNFα gene expression. Interestingly, both inhibition of PC-PLC and PKC clearly decreased LPS-induced IL-8 and TNFα gene expression in PMNs (Fig. 7) thus suggesting that PC-PLC and PKC play pivotal roles in the bacteria-induced dual response of PMN survival and pro-inflammatory response. To verify whether the signal molecules identified to participate in *Y. pseudotuberculosis*-mediated survival of PMNs mirror a general signal transduction pathway for PMN longevity in response to bacteria, cells were exposed to the previously used tyrosine kinase, PI3K, MAPK, PKC or PLC inhibitors prior infection with *S. aureu*s (Fig. 8). As seen for infection with *Y. pseudotuberculosis*, *S. aureus*-mediated repression of caspase 3 activity was independent of tyrosine kinases and p38 MAPK (Fig. 8A), but was strictly dependent on PC-PLC and PKC (Fig. 8B–C). Moreover, consistent with the data on *Y. pseudotuberculosis*, PI3K, and to a less extent p44/42 MAPK and JNK were identified to be partially involved in the *S. aureus*-induced extension of PMN life span (Fig. 8A), hence, indicating a general signaling mechanism for induction of PMN survival in response to bacteria.

## Discussion

This study illustrates how sensing bacteria via TLRs triggers a general mechanism in human PMNs resulting in inhibition of apoptosis, promoting prolonged life span and pro-inflammatory response. We show here that this dual response is mediated by TLR2 and/or TLR4 activation and requires signal transduction via PC-PLC, PKC and to some extent PI3K. Furthermore, the bacteria-stimulated PMN survival depends on the *de novo*-synthesis of survival factors, which goes along with the concomitant generation of pro-inflammatory mediators TNFα and IL-8 and subsequent amplification of the inflammatory response.

Our results show that the anti-apoptotic effect does not depend on actively secreted proteins such as the virulence associated Yop effectors. It has been previously shown that virulent *Y. pseudotuberculosis* is capable of killing RAW264.7 macrophages as well as bone marrow-derived macrophages by inducing apoptosis in a YopJ-dependent manner, through interference with NFκB signaling [13]. YopJ does not have a similar effect on PMNs, since our analyses show activation of NFκB in response to infection with both the virulent and the avirulent strain (data not shown). It can be speculated that *Yersinia* benefits from stimulating PMN survival, and instead directs its pathogenic mechanisms towards other signaling axes in PMNs. One of these signaling axes is for example the pathway involving tyrosine phosphorylation of SLP-76 and SKAP-HOM, targeted by YopH *in vivo* as recently demonstrated by J. Mecsas and coworkers [34]. The SLP-76 associated axis mediates degranulation and phagocytosis, and by blocking this *Yersinia* can survive extracellularly without provoking PMN death and associated clearance mechanisms by macrophages.

The identified pro-survival mechanism was found to be a general effect induced in PMNs upon interaction with different types of bacteria. Furthermore, pre-infection with bacteria also rescued PMNs from Fas/CD95 receptor–induced apoptosis. In analogy with this, priming with granulocyte- colony stimulating factor, GM-CSF, interferon γ or TNFα has been shown to reduce Fas-mediated PMN apoptosis [35], thus suggesting a dominant role of the survival signal over the receptor-death signal. Interestingly, inflammatory PMNs have been shown to be resistant to FAS receptor ligands [36], and it is likely that the resistance to Fas-induced apoptosis observed in this study involves similar mechanisms. In addition, we showed that inhibition of translation by cycloheximide reduced bacteria-induced longevity of PMNs, suggesting that *de novo*-synthesis of signal molecules is required for the extended PMN life span, a finding consistent with constitutive presence of pro-apoptotic mediators in PMNs [37]. PMNs were previously described as terminal cells with low transcriptional and translational capacity, but it became obvious that they exhibit functions next to phagocytosis and elimination of microorganisms. These cells likely play a role in regulating the local immune response by producing inflammatory mediators [1]. In line with this, bacteria-stimulated longevity of PMNs was shown to be accompanied by the production of pro-inflammatory cytokines, including IL-8 and TNFα that stimulates a chemotactic response of uninfected PMNs. Therefore, an induced delay of PMN apoptosis may be an important step, enabling these cells to amplify and fulfil their function in combating bacteria. Enhanced release of cytokines and chemokines does not only play a role in recruiting immune cells to the site of infection but may also act via autocrine mechanisms on inflammatory cells to potentiate defence functions [38], [39].

To date, details of signaling mechanisms that regulate PMN activation and longevity are poorly understood. Only a few studies report on signal transduction of PMNs in response to external survival stimuli whereby GM-CSF-mediated signaling has been most extensively investigated. The intracellular signaling pathway mediating the extended life span of PMNs in response to bacteria identified in the present study is distinct from the mechanism mediated by GM-CSF. Bacteria-induced suppression of PMN apoptosis involves TLR2 or TLR4 ligation and does not depend on tyrosine kinase signaling, which is required for survival induced by GM-CSF [22]. Additionally, our results show that active PC-PLC but not PI-PLC is required for induction of survival. Generally, little is known about the function of PC-PLC in PMNs. This enzyme cleaves phosphatidylcholine, leading to formation of phosphocholine and DAG [40] but the connection between TLRs and PLC is currently less clear. However, PLC has been linked to TLR signalling. It has been reported that TLR4 in synergy with the triggering receptor expressed on myeloid cells 1 (TREM-1) initiates the oxidative burst of PMNs via activation of PLC, PI3K and p38 MAPK [41]; whether this PLC involvement is based on PC-PLC or PI-PLC activity remains to be elucidated. Next to PC-PLC, we found that bacteria-induced PMN survival involves activation of PKC. Participation of PKC in regulation of apoptosis has been suggested in several reports either as a mediator of activation or inhibition [42], [43]. Our analyses using specific pharmacological PKC inhibitors, with consideration of DAG-generating PC-PLC, excluded classical and atypical DAG-independent PKC isoforms in bacteria-mediated delay of PMN apoptosis. Thereby these analyses point to an involvement of a novel PKC isoform, under which PKCε is a putative candidate, which could not be investigated further due to the lack of specific pharmacological tools. This isoform that requires DAG for activation is described to promote cell survival in other systems, including different tumour cells [44] but its role in PMNs is insufficiently understood. Noteworthy, PKCε deficient mice, although having normal thymocyte apoptosis, T cell proliferation and B cell function, show defects in macrophage LPS responses due to aberrant activation of NFκB [45], [46]. Even more interesting in the context of the present study is that PKCε-deficient mice failed to clear infections by both Gram-negative and -positive bacteria [47], thus, hypothesizing that this can be also linked to the lack of survival and pro-inflammatory responses of PMNs. Furthermore, PKCε has been shown to participate in TLR signaling via receptor-associated MyD88 (myeloid differentiation primary response 88) whereby phosphorylated PKCε binds to the regulatory protein 14-3-3β which is required for subsequent gene induction [48]–[50].

**Figure 9:**
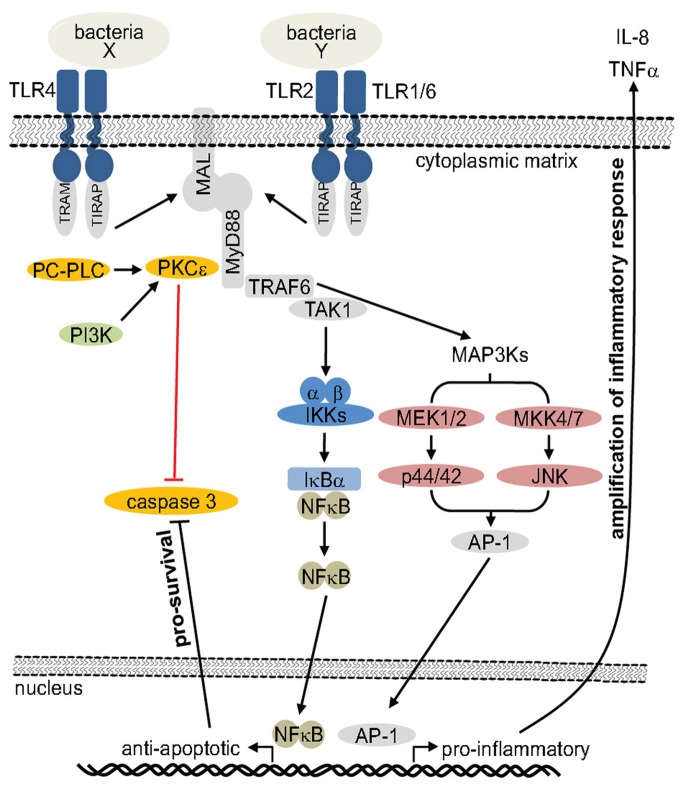
Hypothetical model of signal transduction regulating bacteria-induced survival of PMNs. TLR ligation by bacteria or bacterial products leads to activation of PKCε. Hypothetically PKCε becomes phosphorylated and binds 14-3-3β in a MyD88-dependent manner. Activation of PKCε can occur via PI3K or PC-PLC that provides the PKCε-required DAG. PC-PLC and PKCε transfer signal through a so far unidentified pathway potentially independent of TRAF-6, but dependent on *de novo*-protein synthesis. Simultaneously, p44/42 MAPK and JNK are activated via TRAF6 and provoke translocation of activator protein 1 (AP-1) to the nucleus. TRAF6 also activates NFκB by IκB degradation. The resulting AP-1−/NFκB-induced transcription and production of anti-apoptotic and pro-inflammatory proteins, and the inactivation of proapoptotic proteins, together contribute to suppression of caspase 3 activity. Arrows mark activation, while marks inactivation of target proteins. Black labels are well documented events while red labels are hypothetical. IKK: IκB kinase; IκBα: inhibitor of NFκB; MAL: MyD88 adaptor-like; MEK: mitogen-activated protein kinase kinase; MKK: mitogen-activated protein kinase kinase; TAK1: transforming growth factor β-activated kinase 1; TIRAP: toll-interleukin 1 receptor (TIR) domain-containing adaptor protein; TLR: Toll-like receptor; TRAF-6: TNF receptor associated factor 6; TREM-1: triggering receptor expressed on myeloid cells 1.

In addition to the dominant roles of PC-PLC and PKC in TLR-mediated PMN survival, our data suggest involvement of PI3K acting independently of Akt. Delayed PMN apoptosis stimulated by GM-CSF or IL-8 has been reported to activate Akt resulting in phosphorylation of the pro-apoptotic Bcl-2-associated death promoter (BAD) protein [51]. Moreover, we observed that JNK and p44/p42 MAPK, but not p38 MAPK, may contribute to the bacteria-reduced caspase 3 activity. This is similar to that reported for GM-CSF-induced PMN survival that involves p44/42 MAPK, but not p38 MAPK activation [51]. Noteworthy, the partial effect of inhibiting PI3K, as well as the minor effects of inhibiting p44/42 MAPK or JNK on bacteria-induced PMN survival, compared to the dominant effects by inhibiting PKC or PC-PLC inhibition, suggests involvement of additional PLC/PKC downstream mechanisms promoting PMN survival (Fig. 9). Interestingly, we found that PC-PLC/PKC signaling is not only required for PMN survival but also directly involved in transcriptional regulation of the pro-inflammatory mediators IL-8 and TNFα indicative of a key role of PC-PLC and PKC in a bacteria-induced dual response of PMNs.

In summary, our study demonstrates that bacteria induce prolonged survival of human PMNs via a general mechanism. We provide new insights about a survival-promoting mechanism in PMNs that is initiated via TLR2 or TLR4 ligation and involves PC-PLC and PKC. We show that this bacteria-induced signaling triggers dual functions, increased survival and amplification of the inflammatory response, both effects depending on *de novo*-protein synthesis. Inhibition of apoptosis in response to bacterial infection is likely the first step of PMN activation, and necessary for the establishment of an efficient host defence. Thus, amongst others, host response mechanisms of PMNs, such as generation of pro-inflammatory mediators or reactive oxygen production, known to be crucial for defence against microorganisms, underlie activation of PMNs via suppression of apoptotic pathways. Therefore further studies are needed to understand the complex mechanisms of PMN activation and especially regulation of cell survival beneficial for the host outcome.

## Supporting Information

Figure S1 **(Related to** **Figure 2** **)** *Y. pseudotuberculosis* infection prevents SuperFasLigand-induced loss of mitochondrial potential. PMNs were left untreated or infected with YPIIIpc or pIB102 and treated with the apoptosis inducers for 12 h as described in Fig. 2. The mitochondrial potential was determined by flow cytometry using 1,1′,3,3,3′,3′-hexamethylindodicarbo-cyanine iodide (DiIC1(5)). Carbonyl cyanide 3-chlorophenylhydrazone (CCCP) (50 µM) was used as positive control of low mitochondrial potential. One experiment representative of 4 performed is shown.
(PDF)

Figure S2 **(Related to** **Figure 3** **)** Bacteria-induced PMN survival is mediated by TLR2 and TLR4. (A) PMNs were infected with YPIIIpc or pIB102 at MOI 10∶1 for 30 min or simultaneously stimulated with 1, 10 and 100 ng/ml Kdo2-lipid A or Pam_3_CSK_4_ followed by an incubation for 1, 3, 6 and 12 h. Caspase 3 activity in cell lysates was determined. Data are presented as mean with SEM (N≥3). (B) PMNs remained untreated or were incubated with 10 ng/ml ultrapure LPS or Kdo2-lipid A or 100 ng/ml Pam_2_CSK_4_ or Pam_3_CSK_4_ for 12 h. The mitochondrial potential was determined by using DiIC1(5) and analysis via flow cytometry. One experiment representative of 4 performed is shown. (C) PMNs were infected with YPIIIpc or pIB102 at MOI 10∶1 for 30 min or simultaneously stimulated with 1, 10 and 100 ng/ml ultrapure LPS, Kdo2-lipid A, Pam_2_CSK_3_ or Pam_3_CSK_4_ followed by an incubation for 1, 3, 6 and 12 h. Caspase 8 activity in cell lysates was determined. Data are presented as mean with SEM (N≥3).
(PDF)

Figure S3 **(Related to** **Figure 5** **and** **6** **)** Bacteria-induced PMN survival is independent of tyrosine kinases and Akt but requires PC-PLC and PKC. (A) PMNs were treated with 25 µM genistein for 1 h followed by stimulation with 20 ng/ml GMCSF for 12 h. (B) PMNs were infected with pIB102 at MOI 10∶1 for 10, 20, 30, 45 and 60 min. Protein extracts were subjected to Western blot analysis and probed with antibodies against phosphorylated Akt and total Akt, respectively. One experiment representative of three performed is shown. (C-D) PMNs were treated with 1 µM MK-2206, 10 µM GDC-0086 (C) 1 µM Gö 6876, 1 µM BIM, 1 µM CGP 53353, 10 µM rottlerin, or 1 µM Gö 6983 (D) for 1 h followed by 30 min infection with YPIIIpc or pIB102 at MOI 10∶1 and incubation for 12 h. Caspase 3 activity in rate of FU is indicated. Data are presented as mean with SEM (N = 4); ***p<0.001 compared to 1 h control; *+++p*<0.001 compared to 12 h control.
(PDF)

Figure S4 *S. aureus* induces prolonged PMN survival is independent of PKC-α, -β, -γ, -δ and –ζ. PMNs were pretreated with 1 µM Gö 6976, 1 µM BIM, 1 µM CGP 53353, 10 µM rottlerin or 1 µM Gö6983 for 1 h followed by 30 min infection with *S. aureus* strain Newman at MOI 10∶1 and an additional incubation for indicated time points in gentamicin-containing medium. Caspase 3 activity of cell lysates in rate of FU was determined. Data are presented as mean with SEM (N = 4); ***p<0.001 compared to 1 h control unless indicated differentially; +++*p*<0.001 compared to 12 h control.
(PDF)

Table S1 **(Related to** **Figure 2** **)** Measurement of human PMN cell death. PMNs were either left untreated or infected with YPIIIpc or pIB102 *Y. pseudotuberculosis* strains for 30 min and subsequently treated with staurosporine (STS), SuperFasLigand (SFL) or TNFα and cycloheximide (CHX) for 12 h or were stimulated with LPS, Kdo2-lipid A, Pam_2_CSK_4_ or Pam_3_CSK_4_ for 12 h. States of cell death were monitored by measuring annexin V binding to phosphatidylserine and propidium iodide (PI) binding to DNA. Cell that are negative for both dyes, are termed as healthy cells, annexin V positive cells as apoptotic cells and annexin V and PI positive cells as late apoptotic. Mean and SEM (N≥4) are indicated.
(PDF)
