# Supplementary material for: Bacteria Induce Prolonged PMN Survival via a Phosphatidylcholine-Specific Phospholipase C- and Protein Kinase C-Dependent Mechanism
Source: PLoS One. 2014 Jan 31;9(1):e87859. doi: 10.1371/journal.pone.0087859 (PMC3909253; doi:10.1371/journal.pone.0087859)

Supporting information, Figure S1 (Related to Figure 2)

Y. pseudotuberculosis infection prevents SuperFasLigand-induced loss of mitochondrial potential. PMNs were left untreated or infected with YPIIIpc or pIB102 and treated with the apoptosis inducers for 12 h as described in Fig. 2. The mitochondrial potential was determined by flow cytometry using 1,1′,3,3,3′,3′-hexamethylindodicarbo-cyanine iodide (DiIC$_1$(5)). Carbonyl cyanide 3-chlorophenylhydrazone (CCCP) (50 µM) was used as positive control of low mitochondrial potential. One experiment representative of 4 performed is shown.

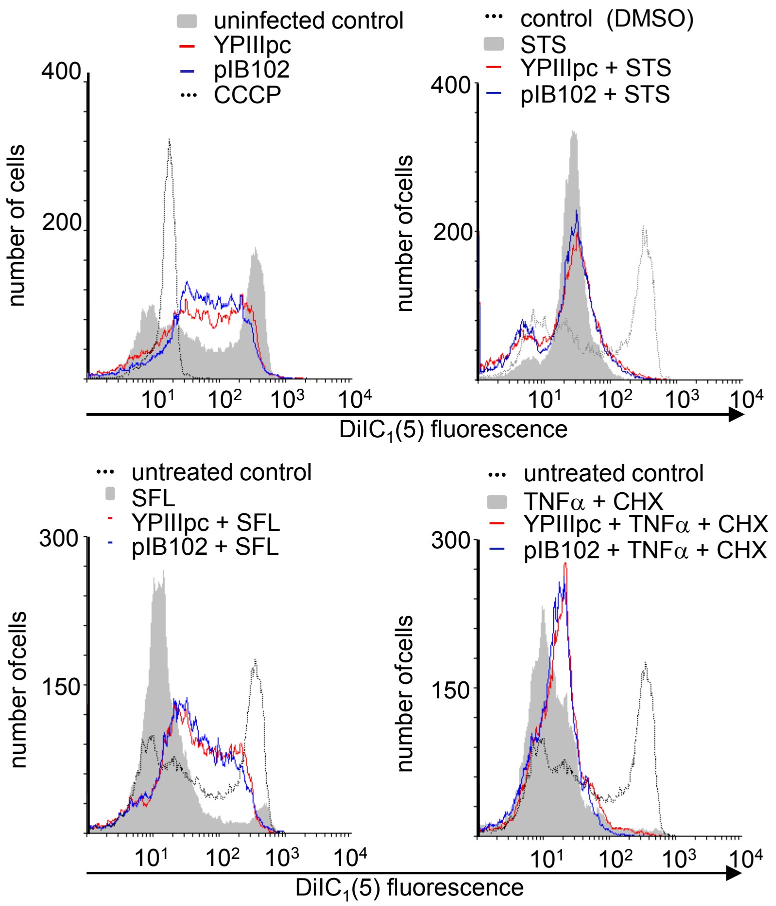

Supplement: Figure S1 — (Related to Figure 2 ) Y. pseudotuberculosis infection prevents SuperFasLigand-induced loss of mitochondrial potential. PMNs were left untreated or infected with YPIIIpc or pIB102 and treated with the apoptosis inducers for 12 h as described in Fig. 2. The mitochondrial potential was determined by flow cytometry using 1,1′,3,3,3′,3′-hexamethylindodicarbo-cyanine iodide (DiIC1(5)). Carbonyl cyanide 3-chlorophenylhydrazone (CCCP) (50 µM) was used as positive control of low mitochondrial potential. One experiment representative of 4 performed is shown. (PDF) [file pone.0087859.s001.pdf]
