# Supplementary material for: Bacteria Induce Prolonged PMN Survival via a Phosphatidylcholine-Specific Phospholipase C- and Protein Kinase C-Dependent Mechanism
Source: PLoS One. 2014 Jan 31;9(1):e87859. doi: 10.1371/journal.pone.0087859 (PMC3909253; doi:10.1371/journal.pone.0087859)

Supporting information, Figure S2 (Related to Figure 3)

Bacteria-induced PMN survival is mediated by TLR2 and TLR4. (A) PMNs were infected with YPIIIpc or pIB102 at MOI 10:1 for 30 min or simultaneously stimulated with 1, 10 and 100 ng/ml Kdo2-lipid A or Pam_3CSK_4 followed by an incubation for 1, 3, 6 and 12 h. Caspase 3 activity in cell lysates was determined. Data are presented as mean with SEM (N≥3). (B) PMNs remained untreated or were incubated with 10 ng/ml ultrapure LPS or Kdo2-lipid A or 100 ng/ml Pam_2CSK_4 or Pam_3CSK_4 for 12 h. The mitochondrial potential was determined by using $DiIC_1(5)$ and analysis via flow cytometry. One experiment representative of 4 performed is shown. (C) PMNs were infected with YPIIIpc or pIB102 at MOI 10:1 for 30 min or simultaneously stimulated with 1, 10 and 100 ng/ml ultrapure LPS, Kdo2-lipid A, Pam_2CSK_3 or Pam_3CSK_4 followed by an incubation for 1, 3, 6 and 12 h. Caspase 8 activity in cell lysates was determined. Data are presented as mean with SEM (N≥3).

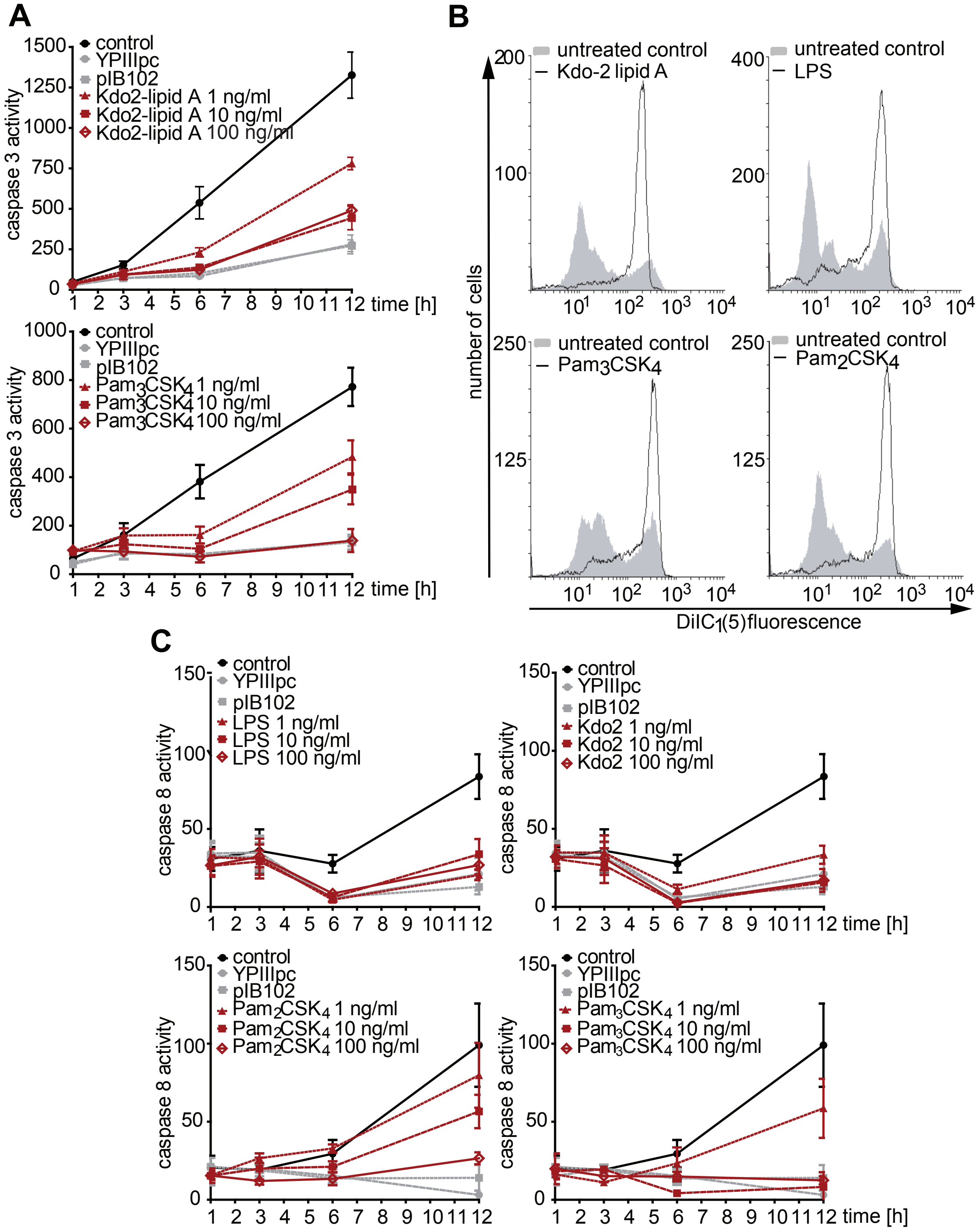

Supplement: Figure S2 — (Related to Figure 3 ) Bacteria-induced PMN survival is mediated by TLR2 and TLR4. (A) PMNs were infected with YPIIIpc or pIB102 at MOI 10∶1 for 30 min or simultaneously stimulated with 1, 10 and 100 ng/ml Kdo2-lipid A or Pam3CSK4 followed by an incubation for 1, 3, 6 and 12 h. Caspase 3 activity in cell lysates was determined. Data are presented as mean with SEM (N≥3). (B) PMNs remained untreated or were incubated with 10 ng/ml ultrapure LPS or Kdo2-lipid A or 100 ng/ml Pam2CSK4 or Pam3CSK4 for 12 h. The mitochondrial potential was determined by using DiIC1(5) and analysis via flow cytometry. One experiment representative of 4 performed is shown. (C) PMNs were infected with YPIIIpc or pIB102 at MOI 10∶1 for 30 min or simultaneously stimulated with 1, 10 and 100 ng/ml ultrapure LPS, Kdo2-lipid A, Pam2CSK3 or Pam3CSK4 followed by an incubation for 1, 3, 6 and 12 h. Caspase 8 activity in cell lysates was determined. Data are presented as mean with SEM (N≥3). (PDF) [file pone.0087859.s002.pdf]
