# Supplementary material for: Bacteria Induce Prolonged PMN Survival via a Phosphatidylcholine-Specific Phospholipase C- and Protein Kinase C-Dependent Mechanism
Source: PLoS One. 2014 Jan 31;9(1):e87859. doi: 10.1371/journal.pone.0087859 (PMC3909253; doi:10.1371/journal.pone.0087859)

Supporting information, Figure S3 (Related to Figure 5 and 6)

Bacteria-induced PMN survival is independent of tyrosine kinases and Akt but requires PC-PLC and PKC. (A) PMNs were treated with 25 µM genistein for 1 h followed by stimulation with 20 ng/ml GM-CSF for 12 h. (B) PMNs were infected with pIB102 at MOI 10:1 for 10, 20, 30, 45 and 60 min. Protein extracts were subjected to Western blot analysis and probed with antibodies against phosphorylated Akt and total Akt, respectively. One experiment representative of three performed is shown. (C-D) PMNs were treated with 1 µM MK-2206 , 10 µM GDC-0086 (C) 1 µM Gö 6876, 1 µM BIM, 1 µM CGP 53353, 10 µM rottlerin, or 1 µM Gö 6983 (D) for 1 h followed by 30 min infection with YPIIIpc or pIB102 at MOI 10:1 and incubation for 12 h. Caspase 3 activity in rate of FU is indicated. Data are presented as mean with SEM (N=4); ****p*<0.001 compared to 1 h control; +++*p*<0.001 compared to 12 h control.

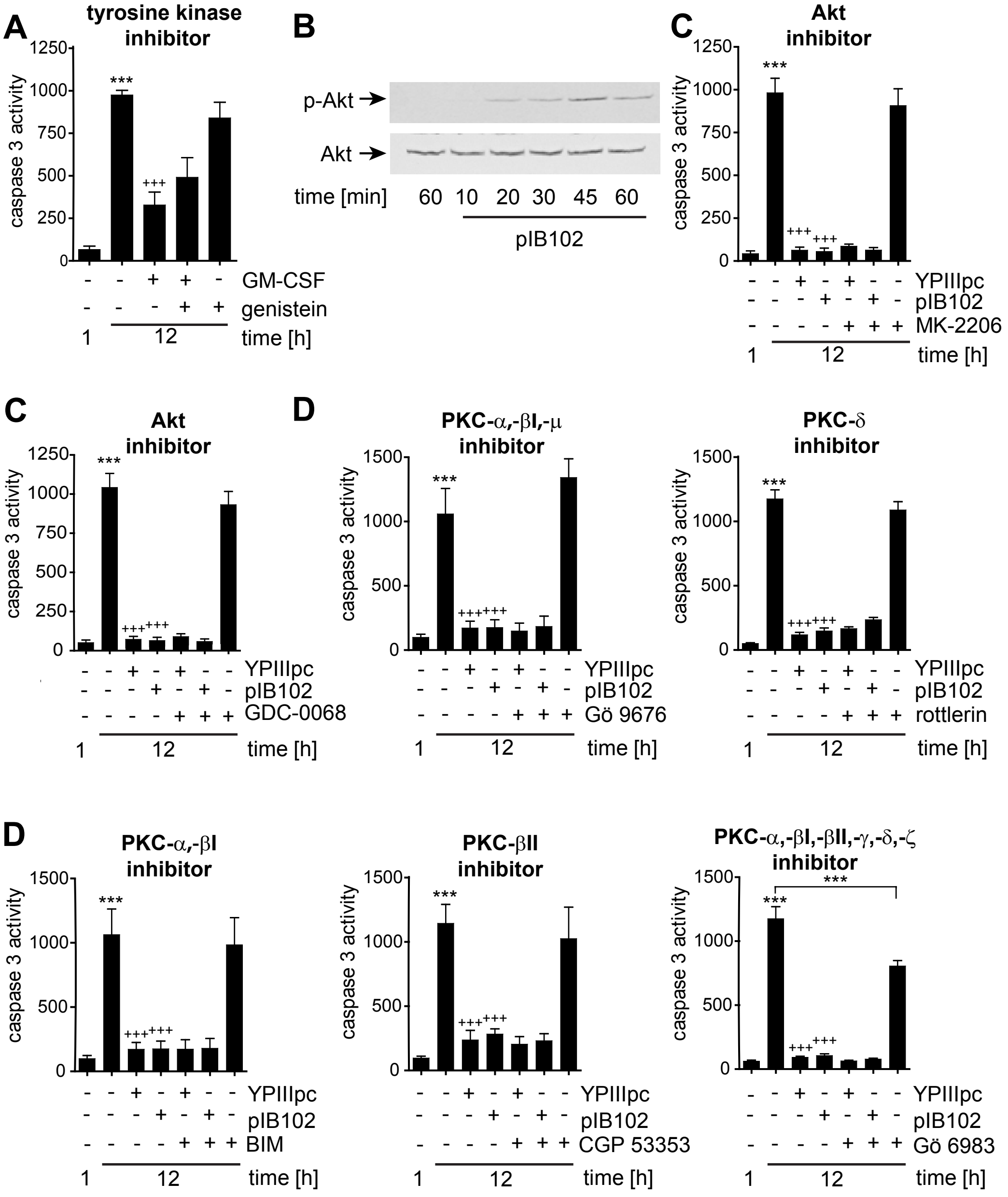

Supplement: Figure S3 — (Related to Figure 5 and 6 ) Bacteria-induced PMN survival is independent of tyrosine kinases and Akt but requires PC-PLC and PKC. (A) PMNs were treated with 25 µM genistein for 1 h followed by stimulation with 20 ng/ml GMCSF for 12 h. (B) PMNs were infected with pIB102 at MOI 10∶1 for 10, 20, 30, 45 and 60 min. Protein extracts were subjected to Western blot analysis and probed with antibodies against phosphorylated Akt and total Akt, respectively. One experiment representative of three performed is shown. (C-D) PMNs were treated with 1 µM MK-2206, 10 µM GDC-0086 (C) 1 µM Gö 6876, 1 µM BIM, 1 µM CGP 53353, 10 µM rottlerin, or 1 µM Gö 6983 (D) for 1 h followed by 30 min infection with YPIIIpc or pIB102 at MOI 10∶1 and incubation for 12 h. Caspase 3 activity in rate of FU is indicated. Data are presented as mean with SEM (N = 4); ***p<0.001 compared to 1 h control; +++p<0.001 compared to 12 h control. (PDF) [file pone.0087859.s003.pdf]
