# Supplementary material for: Bacteria Induce Prolonged PMN Survival via a Phosphatidylcholine-Specific Phospholipase C- and Protein Kinase C-Dependent Mechanism
Source: PLoS One. 2014 Jan 31;9(1):e87859. doi: 10.1371/journal.pone.0087859 (PMC3909253; doi:10.1371/journal.pone.0087859)

Supporting information, Figure S4

S. aureus induces prolonged PMN survival is independent of PKC-α, -β, -γ, -δ and –ζ. PMNs were pretreated with 1 µM Gö 6976, 1 µM BIM, 1 µM CGP 53353, 10 µM rottlerin or 1 µM Gö6983 for 1 h followed by 30 min infection with *S. aureus* strain Newman at MOI 10:1 and an additional incubation for indicated time points in gentamicin-containing medium. Caspase 3 activity of cell lysates in rate of FU was determined. Data are presented as mean with SEM (N=4); ***$p<0.001$ compared to 1 h control unless indicated differentially; +++ $p<0.001$ compared to 12 h control.

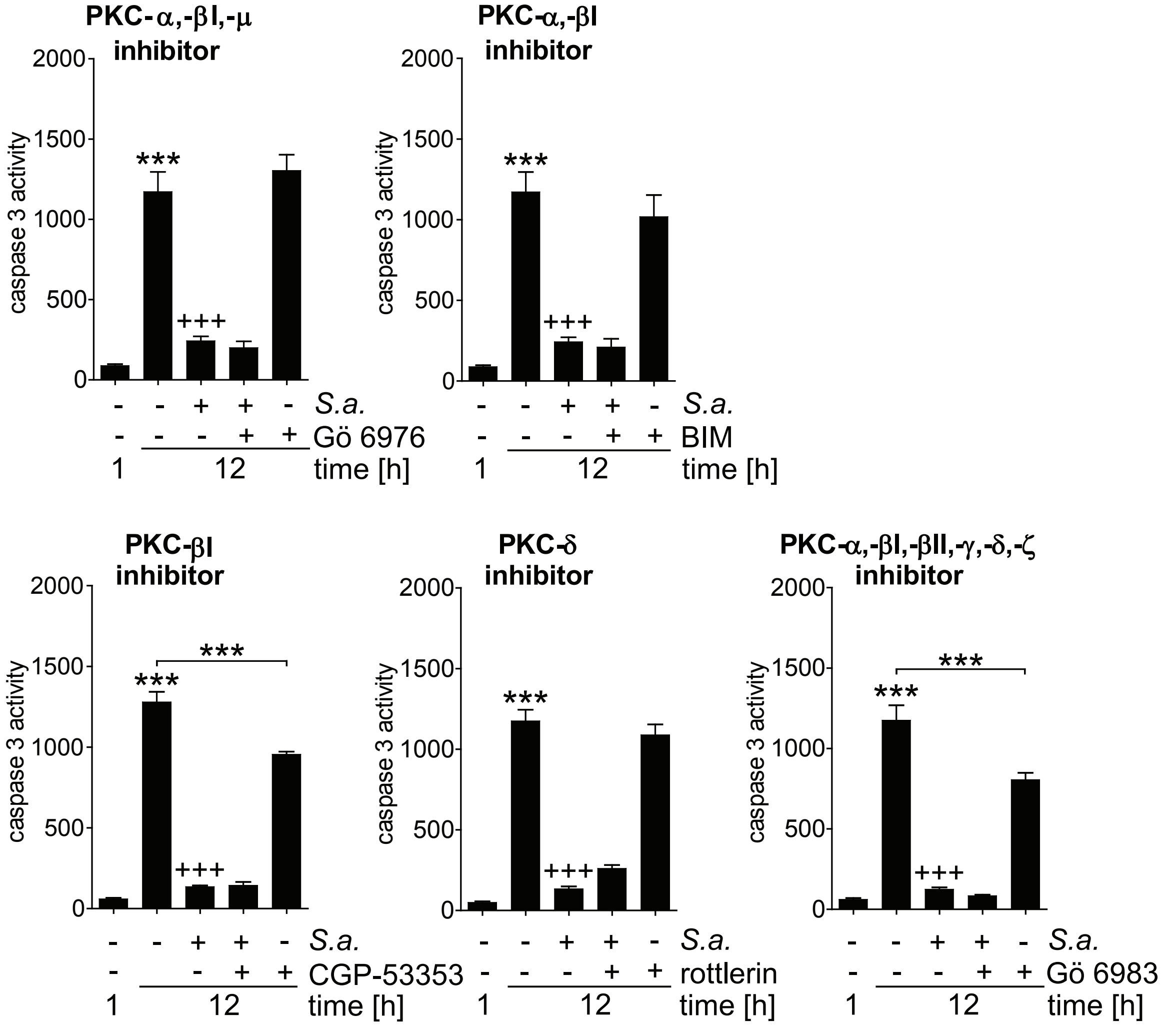

Supplement: Figure S4 — S. aureus induces prolonged PMN survival is independent of PKC-α, -β, -γ, -δ and –ζ. PMNs were pretreated with 1 µM Gö 6976, 1 µM BIM, 1 µM CGP 53353, 10 µM rottlerin or 1 µM Gö6983 for 1 h followed by 30 min infection with S. aureus strain Newman at MOI 10∶1 and an additional incubation for indicated time points in gentamicin-containing medium. Caspase 3 activity of cell lysates in rate of FU was determined. Data are presented as mean with SEM (N = 4); ***p<0.001 compared to 1 h control unless indicated differentially; +++p<0.001 compared to 12 h control. (PDF) [file pone.0087859.s004.pdf]
