# Supplementary material for: Bacteria Induce Prolonged PMN Survival via a Phosphatidylcholine-Specific Phospholipase C- and Protein Kinase C-Dependent Mechanism
Source: PLoS One. 2014 Jan 31;9(1):e87859. doi: 10.1371/journal.pone.0087859 (PMC3909253; doi:10.1371/journal.pone.0087859)
Supplement: Table S1 — (Related to Figure 2 ) Measurement of human PMN cell death. PMNs were either left untreated or infected with YPIIIpc or pIB102 Y. pseudotuberculosis strains for 30 min and subsequently treated with staurosporine (STS), SuperFasLigand (SFL) or TNFα and cycloheximide (CHX) for 12 h or were stimulated with LPS, Kdo2-lipid A, Pam2CSK4 or Pam3CSK4 for 12 h. States of cell death were monitored by measuring annexin V binding to phosphatidylserine and propidium iodide (PI) binding to DNA. Cell that are negative for both dyes, are termed as healthy cells, annexin V positive cells as apoptotic cells and annexin V and PI positive cells as late apoptotic. Mean and SEM (N≥4) are indicated. (PDF) [file pone.0087859.s005.pdf]

Supporting information, Table S1 (Related to Figure 2)

Measurement of human PMN cell death

**stimuli (conc.)**	**healthy cells [%]**		**apoptotic cells [%]**		**late apoptotic cells [%]**	
	mean	SEM	mean	SEM	mean	SEM
control	54.8	1.9	47.7	1.9	1.0	0.1
YPIIIpc MOI 10:1	73.6	1.8	27.2	1.6	3.8	0.4
pIB102 MOI 10:1	70.4	1.6	26.7	1.4	2.6	0.2
control (DMSO)	54.0	1.6	44.1	1.7	1.7	0.1
STS (2 µM)	60.0	2.8	37.5	2.5	2.4	0.3
YPIIIpc + STS	47.2	2.3	49.8	2.4	2.9	0.3
pIB102 + STS	54.5	2.4	42.6	2.4	2.9	0.5
SFL (50 ng/ml)	12.1	1.8	86.1	1.8	3.0	0.5
YPIIIpc + SFL	46.4	2.4	50.3	2.8	3.0	0.4
pIB102 + SFL	46.0	3.6	50.0	3.8	3.7	0.5
TNFα (10 ng/ml) + CHX (5 µg/ml)	18.8	0.9	79.3	1.0	1.9	0.3
YPIIIpc + TNFα + CHX	27.6	2.2	70.0	2.1	2.4	0.2
pIB102 + TNFα + CHX	26.7	2.0	71.5	2.0	1.8	0.1
LPS (10 ng/ml)	84.5	0.8	13.4	0.7	1.9	0.2
Kdo2-lipid A (10 ng/ml)	85.3	0.6	12.0	0.6	2.2	0.1
Pam_2CSK_4 (100 ng/ml)	85.1	0.6	13.3	0.5	1.5	0.1
Pam_3CSK_4 (100ng/ml)	87.6	1.0	10.8	0.7	1.6	0.3

PMNs were either left untreated or infected with YPIIIpc or pIB102 *Y. pseudotuberculosis* strains for 30 min and subsequently treated with staurosporine (STS), SuperFasLigand (SFL) or TNFα and cycloheximide (CHX) for 12 h or were stimulated with LPS, Kdo2-lipid A, Pam_2CSK_4 or Pam_3CSK_4 for 12 h. States of cell death were monitored by measuring annexin V binding to phosphatidylserine and propidium iodide (PI) binding to DNA. Cell that are negative for both dyes, are termed as healthy cells, annexin V positive cells as apoptotic cells and annexin V and PI positive cells as late apoptotic. Mean and SEM (N≥4) are indicated.
